# An interesting diagnosis for a presacral mass: case report

**DOI:** 10.1186/1477-7800-6-18

**Published:** 2009-11-08

**Authors:** Sina Babazadeh, Matthew L Broadhead, John L Slavin, Peter FM Choong

**Affiliations:** 1 Department of Orthopaedics and Department of Surgery, University of Melbourne, St Vincent's Hospital, Melbourne, Australia; 2 Department of Anatomical Pathology, St Vincent's Hospital, Melbourne, Australia; 3 Bone and Soft Tissue Sarcoma Service, Peter MacCallum Cancer Centre, Melbourne, Australia

## Abstract

A presacral mass can present a diagnostic dilemma for the surgical oncologist. Differential diagnoses include congenital causes such as teratoma or chordoma, neurological causes such as neurilemoma or neurofibroma or other malignancies such as lymphoma or sarcoma. Diagnosis usually requires imaging such as CT and MRI and tissue biopsy. We present an unusual cause of a presacral mass being extramedullary haematopoiesis, found incidentally in a 71 year old female. Extramedullary haematopoiesis is defined as the production of myeloid and erythroid elements outside of the bone-marrow. This diagnosis is extremely rare in the presacral area especially in a patient with no haematological abnormalities. A review of the literature is presented.

## Background

Extramedullary haematopoiesis (EH) is defined as the production of myeloid and erythroid elements outside of the bone-marrow. It is usually a mechanism to compensate for haemolytic anaemia such as spherocytosis or thalassaemia, or as a response to abnormal bone-marrow function seen in disorders such as myelofibrosis or leukaemia [1-4].

Approximately only 5% of cases of EH occur outside of the liver and spleen. Of these, the most common sites are surrounding the vertebral column (especially the thoracic region), lymph nodes, retroperitoneum, lung and pleura [1]. Other sites include gastrointestinal tract, brain, kidney and adrenal glands [2,4-6].

The differential diagnosis of a presacral mass can be categorized as congenital (65%), neurogenic (12%), osseous (11%) or miscellaneous (12%). Congenital causes are the most common and include causes such as epidermoid cyst and teratoma, especially in females and chordomas especially in males. Developmental cysts tend to occur in middle-age, are associated with anorectal malformation and sacral bone defects and are generally benign. They are usually asymptomatic, but can become infected, causing pain and discomfort. Other symptoms are associated with mass effect, occasionally causing bowel and bladder symptoms[7]. Teratomas can cause pain and pre-anal drainage in their late stages. In an adult population they are usually benign, as opposed to their paediatric counterpart[7]. Chordomas arise from primitive notochordal remnants. They present with pain and often with bowel and bladder symptoms due to autonomic dysfunction. They grow slowly, but do invade into local bone and soft-tissue. Excision is the mainstay of treatment.

Neurogenic causes of a presacral mass include neurilemomas (Schwanommas) or neurofibromas. Neurilemomas are rare and present with nonspecific symptoms and radiological findings. They can look cystic on imaging. Full excision is recommended as they have a high recurrence with other forms of treatment. They tend to be benign with minimal metastatic potential[8].

Lymphoma, sarcoma and giant cell tumours account for other more common causes [9]. Solid lesions should arouse suspicion of malignancy [10]. Due to the seriousness of some of these differential diagnoses, correct identification of the pathology is mandatory.

## Case

**Figure 1:**
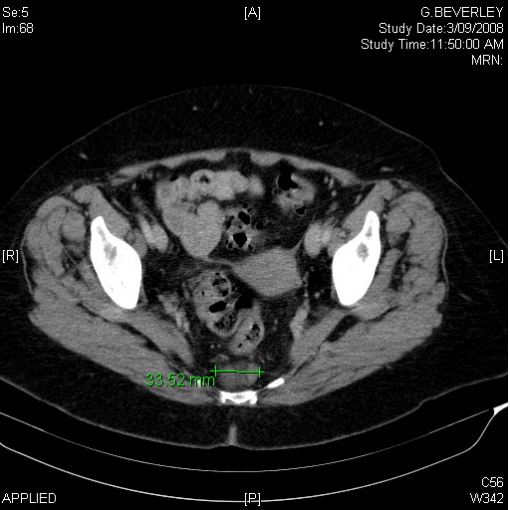
**CT transverse view of lesion showing 33.52 mm diameter lesion in the presacral area**.

**Figure 2:**
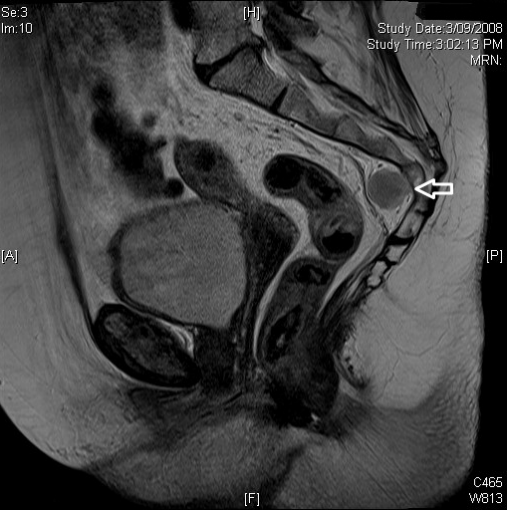
**MRI Sagittal view of lesion**.

A 71 year old female was referred for investigation of a presacral mass found incidentally during a staging CT (figure 1) and MRI (figure 2) of a recently diagnosed rectal carcinoma. The mass was located anterior to S4 and S5 in the presacral space. She was completely asymptomatic of this mass. Except for the recently diagnosed rectal carcinoma and hypertension, she had no other medical issues.

Her CT scan revealed a suggestion of an ill-defined presacral mass, reported as possible oedema. The subsequent staging MRI clarified a 3.3 × 1.9 × 2.3 cm presacral mass at the level of S4/S5. Evidence of bony erosion could not be seen.

**Figure 3:**
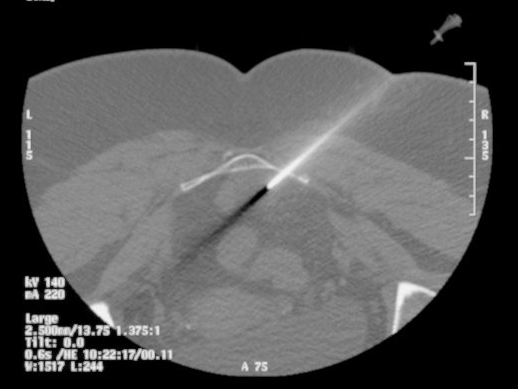
**CT guided biopsy through presacral lesion using posterolateral approach**.

**Figure 4:**
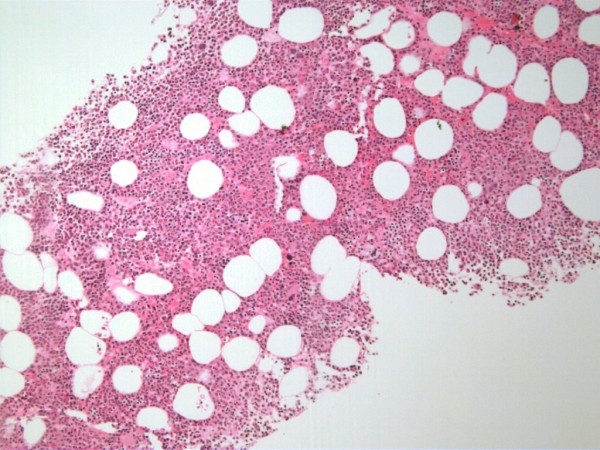
**Histological findings of extramedullary haematopoiesis on biopsy**.

**Figure 5:**
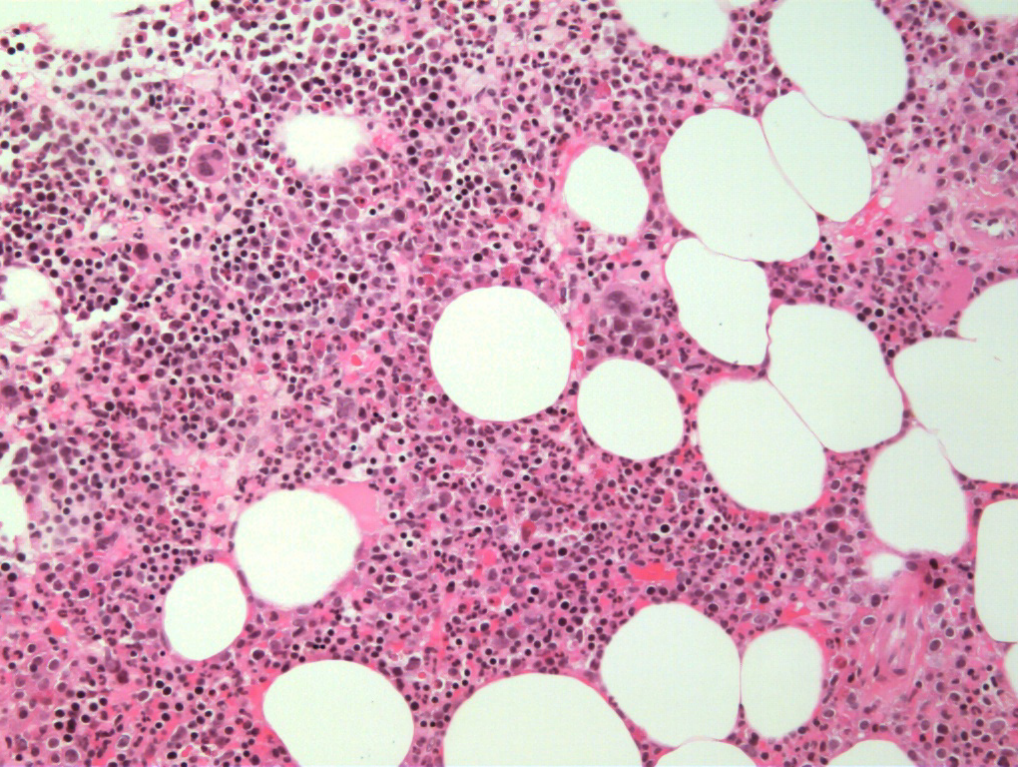
**Fat and haematopoietic tissue consistent with extramedullary haematopoiesis**.

Initially, differential diagnoses for this mass included chordoma, schwannoma and plasmacytoma. The mass was further investigated with a whole body thallium study and a CT-guided biopsy (figure 3). The thallium study revealed no evidence of a thallium avid tumour in the sacrum or elsewhere. The biopsy revealed fat and haematopoietic tissue, consistent with a diagnosis of extramedullary haematopoiesis (figure 4 and 5). The occurrence of EH in the presacral area is an extremely rare finding with only 17 reported cases in the literature.

This remarkable diagnosis was further investigated with the help of a haematologist. A full history found no personal or family history of bleeding disorders or anaemia. Full blood examination and liver function tests revealed no abnormality. A bone marrow biopsy was taken which revealed mildly hypercellular marrow, showing a mild increase in trilineage haemopoiesis. This was likely to be reactive. A cytogenetics study was organized to differentiate reactive marrow from early myelodysplasia. No cytogenetic abnormality was detected.

The case was discussed at a multi-disciplinary meeting and a decision was made that this mass was benign in nature. No further investigations or treatment was deemed necessary. The patient subsequently underwent an uncomplicated resection of her rectal carcinoma. This is only the second case reported in the literature of a prescaral site of extramedullary haematopoiesis with no obvious underlying cause.

## Discussion

In the literature, the majority of the cases of presacral EH were found incidentally (Table 1. Seven of the cases reported where asymptomatic, and were only discovered during investigation of an unrelated disorder [3,5,6,11-13]. Of the rest, four presented with pain [2,14-16], one presented with constipation [17], one presented with symptoms of anaemia [4] and one presented with a pelvic mass [11]. The presenting complaints of the remaining 3 cases could not be identified[18]. A digital rectal exam is very helpful in identifying a presacral mass as a cause of vague symptoms of pain and bowel and bladder dysfunction. Almost all presacral lesions causing symptoms can be detected in this way[7,9].

**Table 1:** Patient list: cases of presacral extramedullary haematopoiesis reported in the literature.

**Author**	**Year published**	**Age of patient**	**Symptoms**	**Diagnosis**	**Predisposing factors**	**Management**	**Outcome**
Babazadeh	2009	71 F	None, found incidentally	CT, MRI, Biopsy (Bx)	None identified	Conservative	No change

Gupta [3]	2008	79 M	None	CT, MRI	Spherocytosis	Conservative	No change after 3 years

Saur [4]	2007	83 F	Anaemia	CT, Bx	Myelofibrosis	Conservative	Not known

Forster [13]	2006	75 F	None, found incidentally	CT, MRI, diagnostic excision	Sacral fracture	Excision	Not known

Youngster [16]	2006	48 F	Painful defecation for 3 months	CT, MRI, diagnostic excision	Beta thalassaemia intermedia	Excision	Symptom free at 12 months

Al-aabassi [6]	2005	23 F	None, found incidentally	US, CT, MRI, TC99, Bx	Beta thalassaemia major,	Regular blood transfusions	Not known

Sarmiento [5]	2003	69 M	None, found incidentally	CT, Bx	Spherocytosis	Conservative	No change at 1 year. Bloods normal

Miyake [12]	2004	54 M	None, found incidentally	CT, MRI, diagnostic excision	Myelofibrosis	Excision	Uneventful

Carazo [2]	1999	60 F	Sciatic pain	CT, MRI, Bx	Mechanical heart valves possibly leading to haemolysis and subsequent anaemia	Conservative	Symptoms improved with time, mass still present at 6 months

Karak [15]	1998	68 F	Lower back pain	TC-99, Bx	None identified	Conservative	Not known

Sporat [14]	1991	Unknown	Pain	CT, Bx	Beta thalassaemia intermedia	Radiotherapy	Good response

Chao [17]	1986	Unknown	Constipation	CT	Beta thalassaemia	Not known	Not known

Sebes [11]	1984	46 F	None, found incidentally	Tc-99, CT	Sickle Cell anaemia	Not known	Not known

Sebes [11]	1984	35 F	Pelvic mass	Tc-99, CT	Beta thalassaemia intermedia	Excision	Not known

A CT-scan is a common preliminary investigation. EH usually appears as heterogeneous lobulated solid mass with smooth margins. Its density is similar to that of soft-tissue, slightly denser than fluid [2,4,16]. Other CT findings that may indicate EH as a cause include hepatomegaly or splenomegaly, consistent with common sites of EH [5,19,20]. Based on CT imaging, a differential diagnosis is lymphoma, which tends to produce a pattern of retroperitoneal involvement of lymph nodes surrounding the great vessels [19,20]. Radiolucency in the sacral area can indicate bony pathology such as osteoid osteoma, chordoma or sarcoma[7].

An MRI of presacral EH often reveals a characteristic well encapsulated tumour with a slightly higher signal intensity than muscle on T1 and T2 weighted imaging, consistent with fatty tissue [2,21]. There is usually no evidence of communication between the mass and neural structures[13]. The mass becomes uniformly enhanced after gadolinium injection [2,16]. Possible iInvolvement of the ureters, rectum and pelvic vessels should be explored when reviewing the MRI.

Nuclear imaging using a whole body scan post intravenous administration of Tc-99m sulfur colloid revealing increased uptake within the site of the mass is also consistent with EH[11,15]. This test can also expose other sites of EH within the body[11]. Angiography can be helpful due to the hypervascularity of these lesions. Homogeneous enhancement post contrast angiography indicates the possibility of EH [22].

With any mass, a tissue sample is usually considered essential to correct diagnosis. The presacral location presents a technical challenge to obtaining such a sample. Possible complications associated with a biopsy of this region include infection and bleeding, especially resulting from insult to the middle sacral artery[10]. Hence, one author suggests that a biopsy should only be attempted in solid lesions with malignant features such as sacral invasion. Biopsy of a cystic lesion has a higher risk of subsequent infection [10]. If a biopsy is to be attempted, the needle tract should be in a position that can be excised en-bloc with the mass if excision is required. Possible routes of access include transgluteal, transrectal, transacral, parasacral and precoccygeal approaches[10]. Histological examination of a biopsy consistent with EH reveals hematopoietic cells with polymorphous infiltrates including megakaryocytes and lymphocytes [4,12,13,15]. Macroscopic features of EH includes a soft, fatty tumour, usually adherent to adjacent structures, revealing an erythematous cut surface [4,12,13,16].

Literature regarding the management of such a condition is limited due to its rarity. Of the reported cases 5 were treated conservatively and monitored, with none reporting an adverse outcome [2-5]. Four patients underwent surgical excision, all reporting good results with no complications [11-13,16]. One patient was treated with regular blood transfusions in an attempt to reduce the need for an extramedullary site for haematopoiesis [6]. One patient was treated with radiotherapy, resulting in symptomatic relief[14]. The management and outcomes of the remaining patients is unclear. Most authors agree that no treatment is necessary unless the patient is symptomatic [3,4,6,12,13,16]. In symptomatic patients, radiotherapy is recommended as a non-invasive and highly effective treatment for EH[1,4]. Other non-invasive forms of treatment include the use of iron chlation with desferrioxamine to relieve anaemia and suppress EH[6], or hydroxyurea to enhance foetal haemoglobin production[23]. Although surgical excision is not usually required in asymptomatic patients, it is often performed due to the sinister nature of the differential diagnoses. Adherence to surrounding tissue can make surgical excision difficult and potentially hazardous.

The prognosis of presacral EH is highly reliant on the underlying cause. All cases with reported follow-up had a satisfactory outcome, with those requiring excision for symptoms remaining symptom free[16], and those treated conservatively, remaining stable[3,5]. One patient who suffered sciatic type pain as a result of the tumour became symptom free within six months, with follow-up exams showing the tumour had decreased in size[2]. A hypothesis explaining this reduction in size is fatty transformation of the mass[24]. Sclerotic tumours may predict a poor prognosis, indicating end-stage myeloproliferative disease[25]. Sacral destruction or symptomatic disease indicates a possible malignant cause and should be investigated fully[26].

## Conclusion

Extramedullary haematopoiesis as a diagnosis for a presacral mass is rare. It is usually asymptomatic and hence typically discovered incidentally. It is heavily associated with haematological disorders, especially myleodysplasia and thalassaemia. A tissue biopsy is generally performed to differentiate this diagnosis from other, more sinister conditions that are more common in this region. Once a diagnosis is made, it is considered safe to treat these lesions conservatively in an asymptomatic patient. In a symptomatic patient, the mass may either be excised or radiotherapy used to shrink its presence.

## Competing interests

The authors declare that they have no competing interests.

## Authors' contributions

SB researched the case and completed a literature review and wrote the initial draft of this case report. MB helped with the literature review and the discussion and edited the final draft of this case report. JS provided invaluable information on the pathology and its significance and was instrumental in describing the pathological findings. PC provided clinical information regarding the patient and the case and edited the final draft of this case report. All authors read and approved the final manuscript.

## Consent

Written informed consent was obtained from the patient for publication of this case report and any accompanying images. A copy of the written consent is available for review by the Editor-in-Chief of this journal.
